# Activity-dependent switches between dynamic regimes of extracellular matrix expression

**DOI:** 10.1371/journal.pone.0227917

**Published:** 2020-01-24

**Authors:** Ivan Lazarevich, Sergey Stasenko, Maiya Rozhnova, Evgeniya Pankratova, Alexander Dityatev, Victor Kazantsev

**Affiliations:** 1 Lobachevsky State University of Nizhni Novgorod, Nizhny Novgorod, Russia; 2 École Normale Supérieure, Paris Sciences et Lettres University, Laboratoire de Neurosciences Cognitives, Group for Neural Theory, Paris, France; 3 Molecular Neuroplasticity Group, German Center for Neurodegenerative Diseases, Magdeburg, Germany; 4 Medical Faculty, Otto-von-Guericke University, Magdeburg, Germany; 5 Center for Behavioral Brain Sciences (CBBS), Magdeburg, Germany; Georgia State University, UNITED STATES

## Abstract

Experimental studies highlight the important role of the extracellular matrix (ECM) in the regulation of neuronal excitability and synaptic connectivity in the nervous system. In its turn, the neural ECM is formed in an activity-dependent manner. Its maturation closes the so-called critical period of neural development, stabilizing the efficient configurations of neural networks in the brain. ECM is locally remodeled by proteases secreted and activated in an activity-dependent manner into the extracellular space and this process is important for physiological synaptic plasticity. We ask if ECM remodeling may be exaggerated under pathological conditions and enable activity-dependent switches between different regimes of ECM expression. We consider an analytical model based on known mechanisms of interaction between neuronal activity and expression of ECM, ECM receptors and ECM degrading proteases. We demonstrate that either inhibitory or excitatory influence of ECM on neuronal activity may lead to the bistability of ECM expression, so two stable stationary states are observed. Noteworthy, only in the case when ECM has predominant inhibitory influence on neurons, the bistability is dependent on the activity of proteases. Excitatory ECM-neuron feedback influences may also result in spontaneous oscillations of ECM expression, which may coexist with a stable stationary state. Thus, ECM-neuronal interactions support switches between distinct dynamic regimes of ECM expression, possibly representing transitions into disease states associated with remodeling of brain ECM.

## Introduction

Understanding the principles and mechanisms of information processing in the central nervous system is among the main objectives of neuroscience. For a long time, the main role in this process was assigned to neurons. Recent studies have shown that, in addition to neurons, an important role in the processing of information also belongs to glial cells and to the ECM [1–4].

The neural ECM, particularly in the form of perineuronal nets, is accumulated during the critical period of postnatal development [4]. Experiences at this time induce the stabilization of functional microcircuits in the brain to support vital brain functions. It is thought that the mature chondroitin sulfate-rich ECM, formed at the end of the critical period, serves as an inhibitory ‘barrier’ that restrains structural plasticity. In a number of experimental studies, it was shown that the ECM molecules in the mature brain are capable of modulating the efficiency of synaptic transmission and neuronal excitability. It is assumed that these mechanisms play a key role in the homeostatic regulation of neuronal activity at relatively long time scales [1,2]. The homeostatic forms of plasticity supported by ECM are thought to prevent pathological hypo- and hyperexcitation of neurons, which may lead to neuronal dysfunction and/or cell death. For example, such a known mechanism as the synaptic scaling allows neurons to maintain neuronal firing rate in a certain range in response to various alterations of afferent inputs or modulation of excitability [5,6]. The synaptic upscaling involves an increase in the concentration of ECM receptors (integrins) on postsynapses, which leads to elevated synaptic expression of AMPA receptors and, hence, to the increased efficiency of excitatory synaptic transmission [1]. Another cascade of regulation involves changing the *Ca*^2+^ influx into neurons through interaction between hyaluronic acid and L-type calcium channels (L-VDCC) [7]. Experimental data demonstrate dynamic changes in cortical and hippocampal ECM during the critical period of development, in response to learning, day-night oscillations in the lateral hypothalamus, long-lasting downregulation of hippocampal and cortical ECM in epilepsy and schizophrenia, and upregulation of ECM in aging, dementia, and depression [1,4,8]. Regulation of ECM concentration is implemented not only via the control of synthesis and secretion of ECM molecules into the extracellular space, but also by the activity of proteases (e.g., tissue plasminogen activator, plasmin, matrix metalloproteinases 2 and 9, aggrecanases 1 and 2, and neurotrypsin), which are released pre- and postsynaptically, as well as from glial cells, to cleave the ECM molecules [4]. As seen in experimental studies on hippocampal interneurons, ECM-neuron interactions involving neuronal Kv channels effectively lead to modulation of the action potential generation threshold, so that a deficit in ECM facilitates firing of interneurons [9–11]. On the other hand, recent experimental findings for pyramidal neurons suggest that fewer spikes are generated after ECM attenuation due to activation of small-conductance calcium-activated potassium (SK) channels [12]. Thus, the considered regulations mediated by the ECM molecules may lead to excitation or inhibition of neuronal activity. In this study we aim to investigate, using a mathematical model of ECM-neuronal interactions, how different regulation mechanisms involved in these interactions shape the dynamics of ECM production and degradation.

A phenomenological model describing the homeostatic regulation of neuronal activity by ECM molecules was first proposed by Kazantsev and colleagues [2]. The model employed a kinetic activation-function description of ECM activity and described the effects of modulation of synaptic transmission and spiking threshold. The activation functions were taken in the simplest possible form reflecting the presence of saturation level what was quite a natural assumption for quasi stationary processes considered at very long time scales. The activation functions are taken in a sigmoidal form [13,14]. The published model predicted the bistability in neuronal firing for particular set of parameters describing ECM-neuronal interaction. In the present work, we further investigated how changes in the polarity of the ECM influence on neurons affects the ECM and neuronal dynamics. In addition to the bistability, the modified model predicted possibility of oscillations. It means that the ECM to neuron crosstalk may induce self-oscillations and, hence, rhythmicity in facilitation/depression of ECM expression at the protein level.

Reductions of the original model of ECM dynamics were done in order to enable the analytical tractability of the resulting model. The polarity of ECM-neuron interactions was changed according to newly available experimental data, showing that that fewer spikes are generated after ECM attenuation due to activation of SK channels [12] compared to the previous model [2]. Importantly, we systematically considered how the prevalence of particular mechanisms of ECM-neuronal interactions might determine the dynamics of ECM concentration levels. We demonstrate that bistability with stable stationary states may be observed regardless of the polarity of ECM influence on neurons—it may be either inhibitory or excitatory. However, in the case when ECM has inhibitory influence on neuronal activity, we predict that bistability is dependent on the activity of proteases, while it is not the case when ECM is excitatory. We show how the excitatory ECM-neuron interaction may lead to spontaneous self-oscillations of ECM molecule concentration, which can coexist with a stable stationary state.

## Methods

### Mathematical model of ECM expression and activity

The processes of ECM synthesis and degradation in a neuronal network are described by the phenomenological approach developed in [2]. The description of neural activity is in accordance with the mean-field Wilson-Cowan type model [15]. Due to the fact that the characteristic timescales of neural dynamics are significantly shorter than those of ECM molecule concentration changes, we set the mean firing rate of the neural population equal to the stationary value, which is a function of the ECM molecule concentration *Q* = *Q*_inf_(*Z*). We assume here a single stationary value of the mean firing rate, e.g. we do not consider bistability induced by E-I interactions in the Wilson-Cowan model [15]. Depending on the polarity of ECM-neuron interactions, the function *Q*_inf_(*Z*) can be either monotonically increasing or decreasing. The key variables describing ECM activity are the ECM concentration *Z*, the concentration of ECM receptors *R*, and the concentration of proteases *P*. The dynamical model consists of the following equations

$$\frac{dZ}{dt}=-\left(\alpha_{Z}+\gamma_{P}P\right)Z+\beta_{Z}H_{Z}\left(Q_{\text{inf}}\left(Z\right)\right)$$ (1)

$$\frac{dP}{dt}=-\alpha_{P}P+\beta_{P}H_{P}\left(Q_{\text{inf}}\left(Z\right)\right)$$ (2)

$$\frac{dR}{dt}=-\alpha_{R}R+\beta_{R}H_{R}\left(Q_{\text{inf}}\left(Z\right)\right)$$ (3)

Here the activation functions *H*_*Z*,*P*,*R*_ all assumed to have a sigmoid shape. An increase in the protease concentration *P* is assumed to be linearly related to the speed of ECM degradation, e.g. *α*_Z*_ = *α*_*Z*_ + *γ*_*P*_*P*. If the ECM-neuronal interactions involve synaptic scaling [16], then stationary neuronal firing rate might also depend on the concentration of postsynaptic ECM receptors. We assume that the resultant extent of the synaptic scaling effect is proportional to the product of ECM molecule concentration and ECM receptor concentration *ZR* since production of ECM molecules and receptors is assumed to be a statistically uncorrelated process. In the case of synaptic scaling, it was shown [2] for a Hodgkin-Huxley-type model that the resultant stationary firing rate *Q*_inf_ can be approximated by a linear function of *ZR*. The timescales of ECM receptor dynamics are at least an order of magnitude shorter than those of ECM molecules and receptors in the original model [2], so that variable *R* can be approximated by its steady-state value *R*_inf_(*Q*). For other ECM-neuron interaction mechanisms there is no dependence on the ECM receptor concentration *R*, as shown in further sections. In any case the dynamical system might be reduced to a two-dimensional one, so that it is rather analytically tractable.

## Results

### ECM bistability

The presented first-order relaxation kinetics model for ECM proteins and proteases concentrations can be viewed as an approximation of a more detailed model of ECM degradation and remodeling (e.g., developed in [17]). To avoid the dynamic effects induced by the incorporation of specific biophysical processes, we only define parameters that have direct biophysical interpretation in relation to the kinetics. These parameters are namely the rate of concentration decay and activity-induced production rate of ECM proteins and proteases.

Let us consider the case when the ECM-neuron interaction feedback loop involves either clustering of Kv potassium channels (inhibitory ECM effect) or inhibition of small-conductance calcium-activated SK potassium channels (excitatory ECM effect). In these cases, ECM-neuron interactions are independent of postsynaptic ECM receptor concentration *R*, since the regulation mechanism involves modulation of somatic membrane receptors of the neuron. Hence, the stationary firing rate of neurons depends only on the ECM concentration.

We assume the effect of ECM concentration on neuronal firing rate might be approximated by a linear dependence *Q*_inf_ = *Q*_0_ + *α*_*Q*_*Z*. This is a fair assumption when the AP firing threshold is being modulated by ECM [2], and we use the same description when neuronal firing is modulated through SK channel activation. We arrive at the following system of equations describing ECM dynamics:

$$\frac{dZ}{dt}=-\left(\alpha_{Z}+\gamma_{P}P\right)Z+\beta_{Z}{\hat{H}}_{Z}\left(Z\right)=\hat{Z}\left(Z,P\right)$$ (4)

$${\hat{H}}_{Z}\left(Z\right)=\left(Z_{0}-\frac{Z_{0}-Z_{1}}{1+\text{exp}\left(K_{Z}^{-1}\left(Q_{0}+\alpha_{Q}Z-\theta_{Z}\right)\right)}\right)$$ (5)

$$\frac{dP}{dt}=-\alpha_{P}P+\beta_{P}{\hat{H}}_{P}\left(Z\right)=\hat{P}\left(Z,P\right)$$ (6)

$$H_{P}(Z)=\left(P_{0}-\frac{P_{0}-P_{1}}{1+\text{exp}\left(k_{P}^{-1}\left(Q_{0}+\alpha_{Q}Z-\theta_{P}\right)\right)}\right)$$ (7)

**Fig 1:**
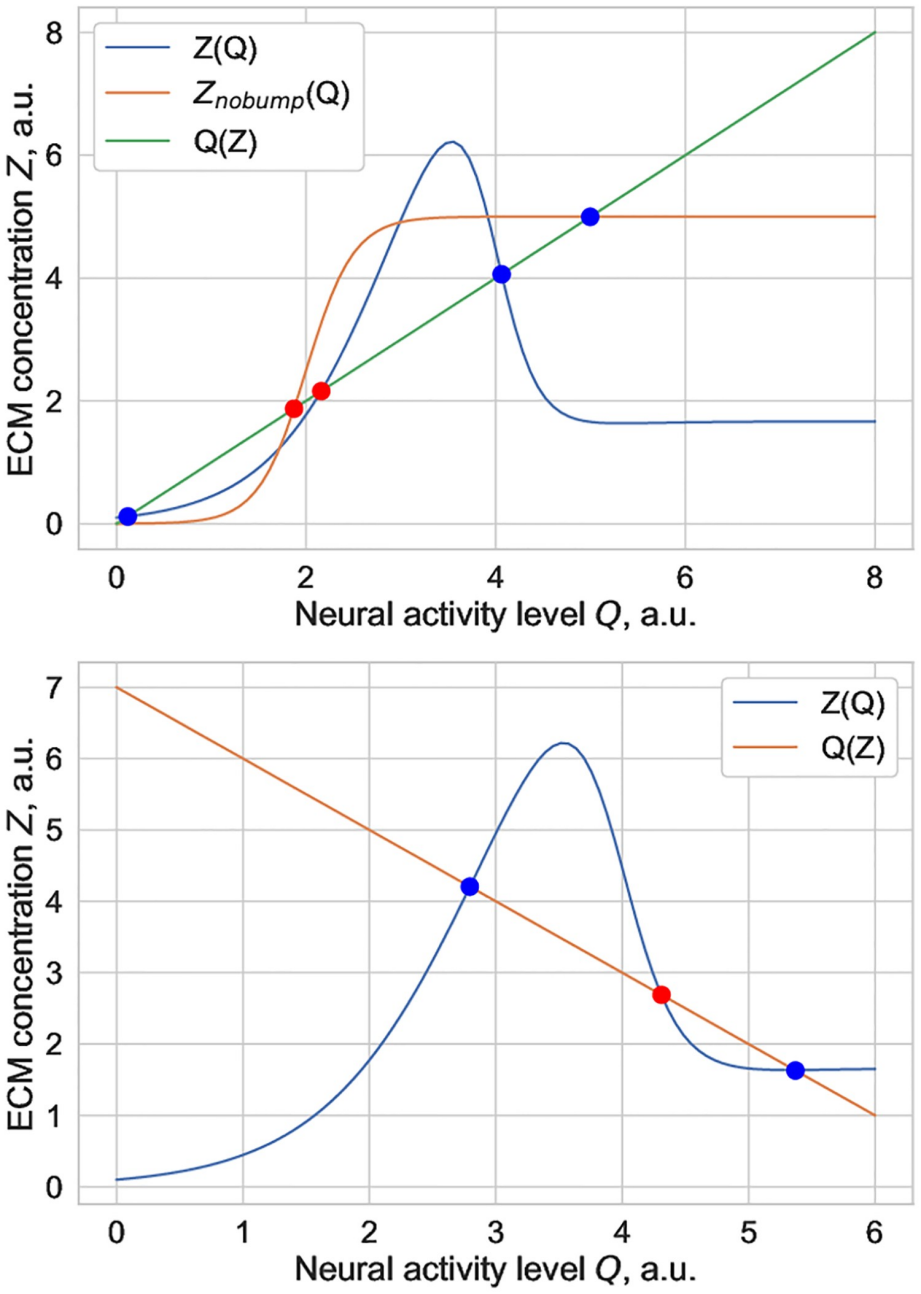
Examples of equilibrium curves corresponding to Eqs (4)–(6) in the (Z, Q) phase plane for the case of (top) excitatory ECM-neuron interaction and (bottom) inhibitory ECM-neuron interaction. Both panels show the existence of bistable solutions regardless of the polarity of ECM-neuron interactions. The intersections of the nullclines determine the equilibria of the system: blue points are stable, red points are unstable.

Let us first qualitatively show that ECM concentration might be bistable in this system regardless of the sign of *α*_*Q*_. The equilibrium curves in the ECM-concentration firing rate phase plane (*Z*, *Q*) are shown in Fig 1.

It is apparent that there are cases of bistability, which correspond to the line *Q*_inf_ = *Q*_0_ + *α*_*Q*_*Z* intersecting the *Z*_inf_ curve in three points, two stable and one unstable stationary solutions, correspondingly. Note that depending on the sign of the *α*_*Q*_ parameter, which controls whether ECM influence on neurons is inhibitory or excitatory, the bistability effect is induced by different mechanisms. When the ECM-neuron interaction is excitatory, and hence the slope of the *Q*_inf_(*Z*) line is positive, there can exist bistable solutions regardless of whether the curve *Z*_inf_(*Q*) has a”bump” at intermediate values of *Q*. A monotonically increasing sigmoid form of *Z*_inf_(*Q*) (which corresponds to the absence of protease effect on ECM, e.g. *α*_*P*_ = 0) would be enough to yield a set of bistable solutions. On the other hand, if the ECM-neuron effect is inhibitory (negative *α*_*Q*_), bistable solutions only exist in the presence of the bump in the equilibrium curve *Z*_inf_(*Q*). This bump occurs because when neuronal firing rate *Q* increases, the synthesis of ECM molecules is upregulated, but the concentration of proteases *P* increases as well, though at slightly higher values of the firing rate. An increase in protease concentration *P* leads to ECM degradation, hence the equilibrium value *Z*_inf_ is smaller at higher firing rates compared to the intermediate range of *Q* values. The height of this bump is determined by the strength of protease-induced ECM degradation (value of *α*_*P*_).

In biophysical terms, we predict that if the prevalent regulation cascade determining ECM-neuronal interactions restrains neuronal excitability, then ECM bistability can only be implemented if proteases demonstrate a strong effect on ECM degradation. If ECM-neuronal interactions support neuronal excitability, the bistability effect does not depend on the strength of protease-ECM interaction and might be implemented even in the absence of protease-dependent ECM degradation.

### Homeostatic ECM oscillations

Let us more closely consider the case of excitatory ECM-neuron interactions (*α*_*Q*_ *>* 0), for instance, implemented through inhibition of neuronal SK channels, as seen experimentally. First, let us study the number and stability of equilibrium states of the Eq. system (4)–(7). As free parameter we consider the effective firing rate threshold for ECM production *θ*_*Z*_.

**Fig 2:**
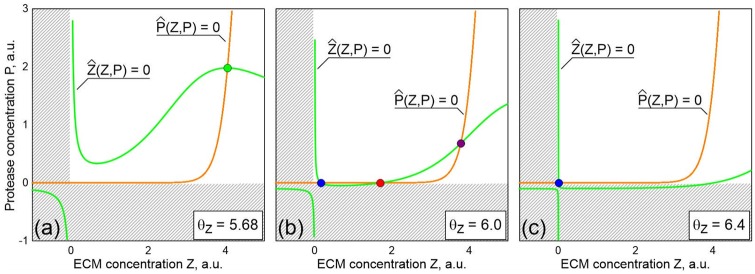
Nullclines *Z*ˆ(*Z*,*P*) = 0 and *P*ˆ(*Z*,*P*) = 0 for three different values of the ECM production threshold *θ*_*Z*_: (a) *θ*_*Z*_ = 5.68, (b) *θ*_*Z*_ = 6 and (c) *θ*_*Z*_ = 6.4. The intersections of the nullclines determine the equilibria of the system: green (focus) and blue (node) points are stable, red (saddle) and purple (focus) are unstable. Biologically meaningless areas are indicated as shaded domains. Other parameters are given in Table 1.

The number of equilibrium points is determined by the number of intersections of the nullclines *Z*ˆ(*Z*,*P*) = 0 and *P*ˆ(*Z*,*P*) = 0. Fig 2 shows the nullcline intersections for three different values of *θ*_*Z*_: *θ*_*Z*_ = 5.68, *θ*_*Z*_ = 6 and *θ*_*Z*_ = 6.4. It is apparent that changes in *θ*_*Z*_ only influence the curve *Z*ˆ(*Z*,*P*) = 0 while the *P*ˆ(*Z*,*P*) = 0 curve stays the same. For *θ*_*Z*_ = 5.68, the intersection of the curves determines the unique equilibrium of the system that is shown as the green point in Fig 2a. As will be shown later, this point is a stable focus. With increasing *θ*_*Z*_, the upper part of the *Z*ˆ(*Z*,*P*) = 0 curve goes down relative to the *P*ˆ(*Z*,*P*) = 0 curve. For *θ*_*Z*_ = 6, three intersection points exist. Particularly, in Fig 2b, two of these points are unstable (the red point is of saddle type, the purple one is an unstable focus) and the blue point corresponds to the stable node. The following increase of *θ*_*Z*_ leads to the case with one intersection of the nullclines. In Fig 2c, this point is shown by blue color. The stability analysis carried out for the linearized equations shows that this is the stable node. Thus for considered set of the parameters, the system (4)–(7) has three equilibria within the interval *θ*_*Z*_ ∈ (*θ*_*Z*_^*L*^, *θ*_*Z*_^*R*^) where the left boundary corresponds to coincidence of blue and red points, while the points denoted by red and purple colors coincide at *θ*_*Z*_ = *θ*_*Z*_^*R*^.

**Table 1:** Parameters of the model (4–7).

Parameter	Values
*Q*_0_[arb.u.], *α*_*Q*_[arb.u.]	5, 0.23
*α*_*Z*_ [ms^−1^], *k*_*Z*_ [arb.u.], *β*_*Z*_ [ms^−1^]	0.0001, 0.15, 0.01
*α*_*P*_ [ms^−1^],*β*_*P*_ [ms^−1^],*θ*_*P*_ [arb.u.],*k*_*P*_[arb.u.]	0.001, 0.001, 6, 0.05

To investigate the stability of these equilibrium points we consider the Jacobi matrix:

$$A=\left[\begin{array}{ll} -\left(\alpha_{Z}+\gamma_{P}P\right)+\varsigma_{1} & -\gamma_{P}Z\\ \varsigma_{2} & -\alpha_{P} \end{array}\right]$$ (8)

where

$$\begin{array}{l} \varsigma_{1}=-\frac{\alpha_{Q}\beta_{Z}\left(Z_{0}-Z_{1}\right)\text{exp}\left(-k_{Z}^{-1}\left(Q_{0}+\alpha_{Q}Z-\theta_{Z}\right)\right)}{k_{Z}{\left(1+\text{exp}\left(-k_{Z}^{-1}\left(Q_{0}+\alpha_{Q}Z-\theta_{Z}\right)\right)\right)}^{2}}\\ \varsigma_{2}=-\frac{\alpha_{Q}\beta_{P}\left(P_{0}-P_{1}\right)\text{exp}\left(-k_{P}^{-1}\left(Q_{0}+\alpha_{Q}Z-\theta_{P}\right)\right)}{k_{P}{\left(1+\text{exp}\left(-k_{P}^{-1}\left(Q_{0}+\alpha_{Q}Z-\theta_{P}\right)\right)\right)}^{2}} \end{array}$$ (9)

with *Z* and *P* taken in the equilibrium point (Z_*_, P_*_) obtained from the following system:

$$\begin{array}{l} \hat{Z}\left(Z_{*},P_{*}\right)=0\\ \hat{P}\left(Z_{*},P_{*}\right)=0 \end{array}$$ (10)

**Fig 3:**
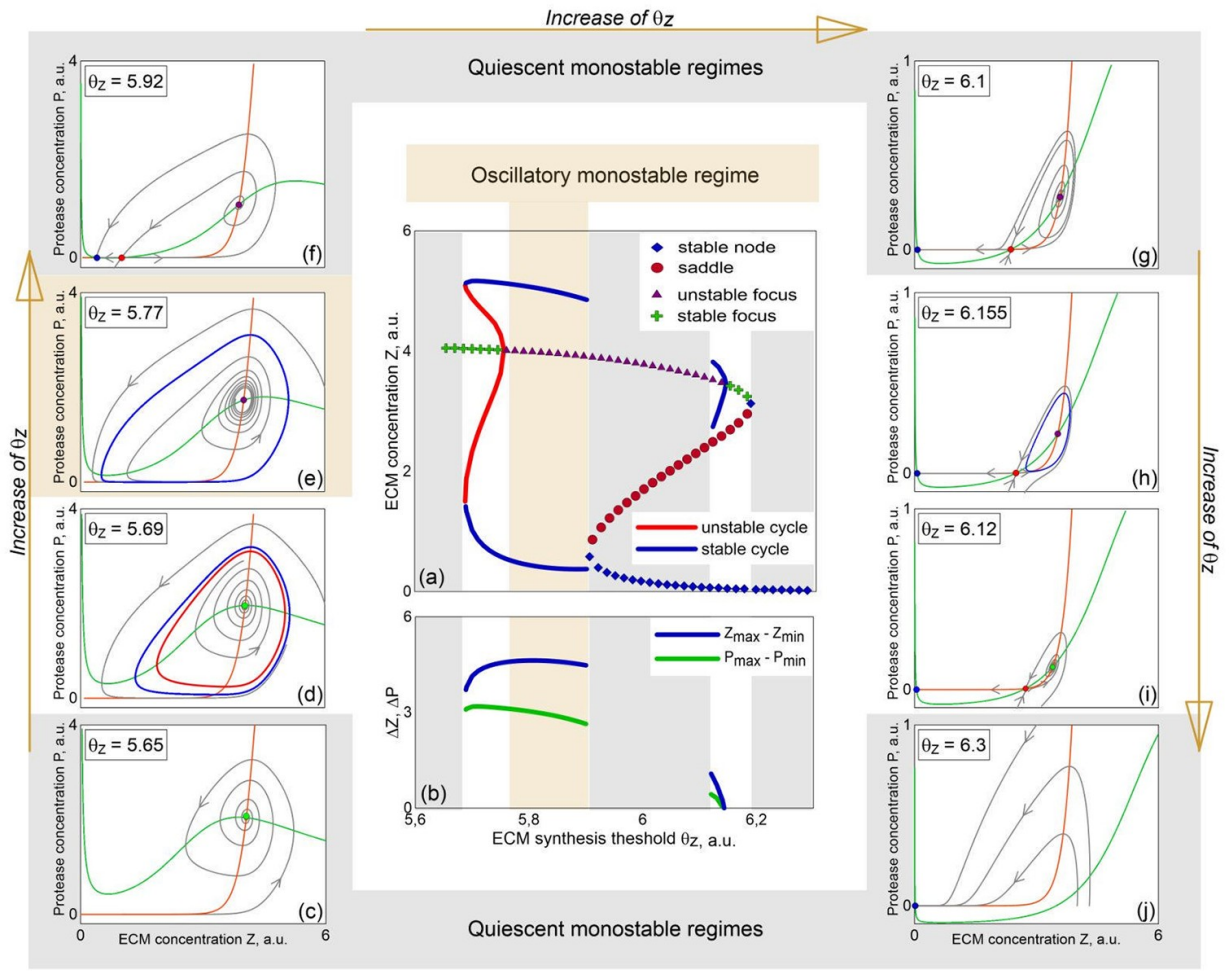
(a) Bifurcation diagram of the system (4)–(7) for varied ECM production threshold value *θ*_*Z*_. (b) Differences *ΔZ = Z*_*max*_*–Z*_*min*_ and *ΔP = P*_*max*_*–P*_*min*_ as functions of *θ*_*Z*_. (c)-(j) Phase portraits of the system for various values of *θ*_*Z*_. Other parameters are given in Table 1.

Finding the eigenvalues of the matrix (8) as the roots *s*_1_ and *s*_2_ of the characteristic equation:

$$s^{2}+[\alpha_{Z}+\alpha_{P}+\gamma_{P}P_{*}-\varsigma_{1}]s+\alpha_{P}(\alpha_{Z}+\gamma_{P}P_{*}-\varsigma_{1})+\gamma_{P}\varsigma_{2}Z_{*}=0$$ (11)

the stability of the equilibria (Z_*_, P_*_) for various *θ*_*Z*_ can be determined. Particularly, in Fig 3a, symbols correspond to the stationary Z_*_ values obtained for various values of the ECM production threshold *θ*_*Z*_. Different types of equilibrium points are denoted by different symbols. In addition to stable stationary states, there might exist oscillatory regimes as well, with corresponding limit cycles in the phase space of the system. Blue curves in Fig 3a demonstrate the minimal *Z*_*min*_ and maximal *Z*_*max*_ values, which *Z* can achieve on the stable limit cycle for different values of *θ*_*Z*_. The red curves denote the same, but for unstable limit cycle. The differences *ΔZ = Z*_*max*_*–Z*_*min*_ and *ΔP = P*_*max*_*–P*_*min*_ for stable limit cycles as functions of *θ*_*Z*_ are presented in Fig 3b.

To discuss the mechanisms of various regimes emergence, we start with the left boundary of the considered interval of *θ*_*Z*_ change. Namely, the phase portrait presented in Fig 3c is obtained for *θ*_*Z*_ = 5.65. This quiescent monostable regime with the unique attracting set being the stable focus is observed for small *θ*_*Z*_ and denoted in Fig 3a by green symbols. For *θ*_*Z*_≈5.685, a stable and an unstable limit cycles appear as a result of a fold-limit-cycle bifurcation. Particularly, the phase portrait for *θ*_*Z*_ = 5.69 with two limit cycles is shown in Fig 3d. In this figure and others, the stable limit cycle is drawn in blue, and unstable limit cycle is in red. Note that for *θ*_*Z*_ = 5.69 we observe the first type of bistability: both the stable focus and the stable limit cycle of large amplitude are co-exist in the phase space of the system. For *θ*_*Z*_ ≈ 5.755, as the result of subcritical Andronov-Hopf bifurcation, unstable limit cycle turns into the equilibrium point, and the stable focus becomes unstable, as shown in Fig 3e. Within the *θ*_*Z*_ ∈ (5.755, 5.904) interval the oscillatory monostable regime is observed with the unique attracting set being the stable limit cycle that disappears through the saddle-node separatrix-loop bifurcation at *θ*_*Z*_ ≈ 5.904. The two equilibrium states that appear in the result of this bifurcation (a stable node and a saddle)”walk away” with increasing *θ*_*Z*_ and, in particular, for *θ*_*Z*_ ≈ 5.92 the phase portrait of the system has a form as shown in Fig 3f. With further increase in the value of *θ*_*Z*_, another limit cycle appears. The mechanism of its appearance is the following: for *θ*_*Z*_ = 6.1 an unstable separatrix bends the stable separatrix outside, Fig 3g; the separatrices get closer with increasing *θ*_*Z*_, and for *θ*_*Z*_ = 6.12 the stable separatrix covers the unstable one. The change in relative position of separatrices is taking place with negative saddle value *σ* = *λ*_1_ + *λ*_2_ *<* 0. Therefore, a stable limit cycle has to appear, which is exactly what is observed: Fig 3h shows the cycle which appeared in the result of saddle separatrix-loop bifurcation with blue color. It is noteworthy that the amplitude of this oscillatory state is rather small (for comparison with the large-amplitude cycle that was observed before, the differences *ΔZ* and *ΔP* are also presented in Fig 3b) and its generation depends on the initial conditions because it co-exists with the stable node in the phase space. This is the second type of bistability: the co-existence of the stable node and the small-amplitude limit cycle that is observed for *θ*_*Z*_ ∈ (6.11, 6.14). For *θ*_*Z*_ ≈ 6.14 the stable cycle turns into the equilibrium point and vanishes through the supercritical Andronov-Hopf bifurcation. The equilibrium point (focus) becomes stable. The transition to the third type of bistability occurs: two types of stationary states (the stable focus and the stable node, Fig 3i) co-exist in the phase space of the system. With further increase of *θ*_*Z*_ the focus turns into a node and disappears in the result of another saddle-node bifurcation at *θ*_*Z*_ ≈ 6.19. For *θ*_*Z*_ > 6.19, the quiescent monostable regime with the stable node being the unique attracting set in the phase space of the system is observed.

In summary, with increasing value of ECM production threshold *θ*_*Z*,_ we can observe two areas in the parameter space of the model, where oscillatory dynamics of ECM concentration levels might occur, either spontaneously (if the limit cycle is the unique attracting set in the phase space) or as a result of external stimulation (if ECM concentration was initially in a stationary state). The nature of these ECM oscillations may be understood qualitatively—an increase in neuronal activity drives an increase in ECM concentration, and further release and activation of proteases that degrade ECM molecules, which in turn lowers neuronal activity. Proteases are less active at low neuronal activity levels, and the positive ECM-neuronal firing feedback loop drives the activity levels up again.

**Fig 4:**
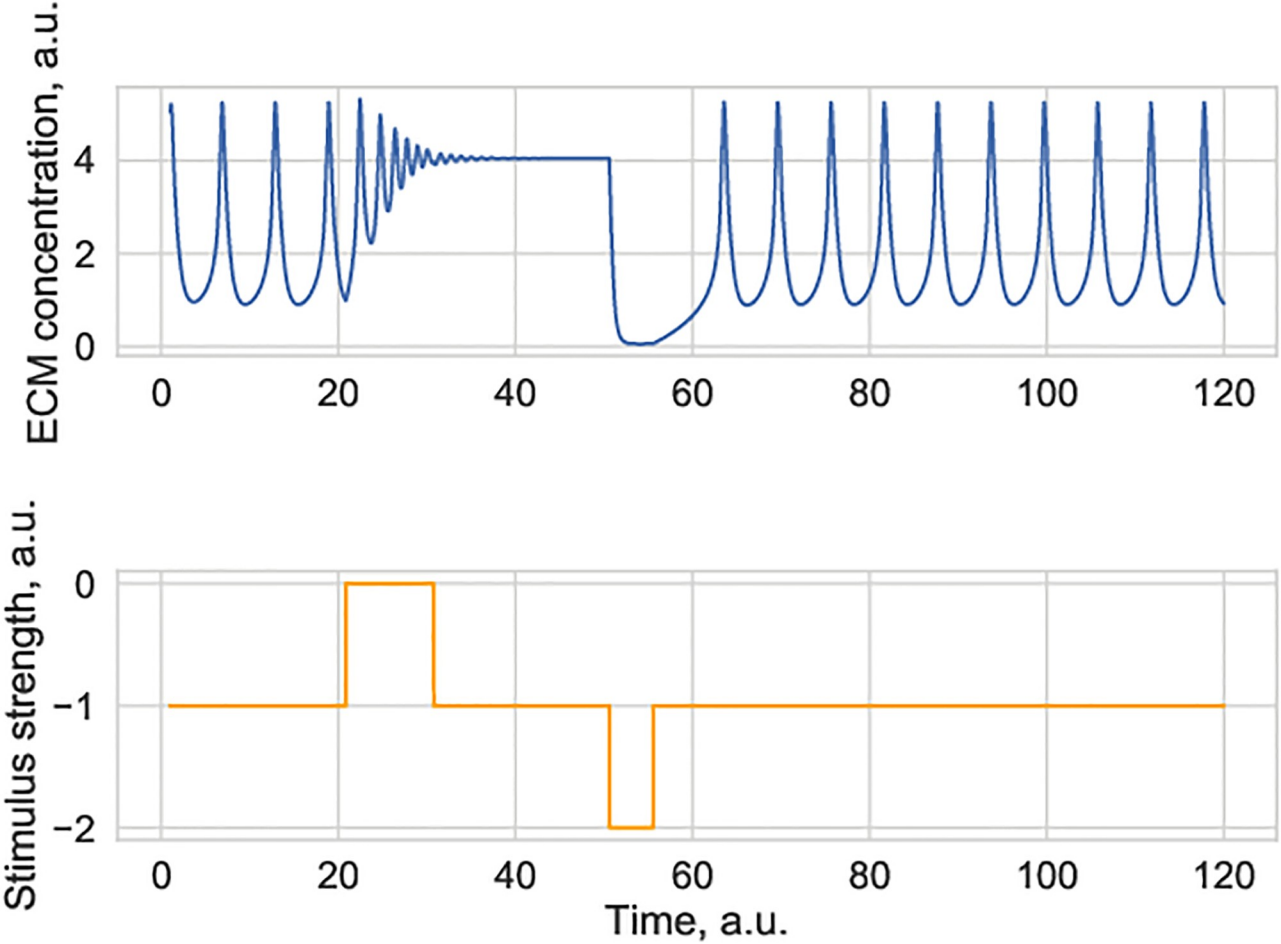
Simulated ECM concentration trace under the conditions when the ECM-protease system exhibits coexistence of a stable limit cycle and a stable stationary state. Application of an external stimulus (e.g. a spontaneous increase or decrease in neural activity) may induce dynamical switches between activity states. This is an imposed change in neural activity. Parameter value for *θ*_*Z*_ = 3.75. Other parameters are given in Table 1.

Fig 4 shows neuronal firing-induced switches between oscillations and a stationary ECM state. Spontaneous changes in the level of neural firing act as an effective stimulus to the ECM-proteases system, which might drive the system away from the locally stable manifold.

The physical timescale values of the observed ECM oscillations are quite extended in our model since the key assumption is that ECM dynamics is much slower as compared to neuronal dynamics. Experimentally observed changes in ECM concentration may be on the timescale of hours to days [1], but the exact relaxation time values in the model remain to be estimated.

### Influence of ECM receptor dynamics

In the case when the prevalent mechanism of ECM-neuron interactions is through synaptic scaling, the dynamics of ECM receptors might influence ECM dynamics in general. As mentioned above, typically the characteristic timescales of ECM receptor dynamics is significantly shorter than that of ECM molecules and proteases, so that *R* can be replaced with the stationary value *R*_inf_(*Q*). The dynamics of ECM receptors is, however, significantly slower than that of neuronal activity, so we can set *R* = *R*_inf_(*Q*_inf_). Assuming that the stationary firing rate level scales linearly with the product *ZR*, we arrive at

$$Q_{\text{inf}}(Z)=\frac{Q_{0}+\alpha_{Q}R_{0}Z}{1+\alpha_{Q}\alpha_{R}Z}$$ (12)

where we also introduced a linear approximation *R* ≈ *R*_0_ − *α*_*R*_*Q*. It is clear that the slope of ECM-neuronal interaction curve is now activity-dependent, decreasing with higher levels of neural activity. This might result in an activity-driven formation of bistable or oscillatory states of the ECM concentration.

Another limit case is when the dynamics of ECM receptors is slow even in comparison to characteristic timescales of ECM remodeling (e.g. the period of ECM concentration oscillations), when the value of *R* ≈ R* is approximately constant on the timescale of interest. In this case the analysis would be the same as in the case of SK-channel mediated ECM-neuron interactions, with negligible activity-dependent changes in the system’s dynamics.

## Discussion

In summary, we have investigated ECM concentration in a mathematical model of ECM-regulated modulation of neural activity. The model is based on the following key assumptions: (a) synthesis of ECM molecules and ECM-degrading enzymes is controlled by the level of neuronal activity, (b) changes in ECM levels may, in turn, modulate neuronal activity, in either excitatory or inhibitory manner, depending on the prevailing mechanism of ECM-neuronal interaction. Mathematically, the model can be reduced to a set of two or three coupled differential equations, depending on the assumptions concerning the nature of ECM-neuronal interactions and characteristic timescales of postsynaptic ECM receptor production. The inhibitory effect of increased ECM levels on neural activity was observed to induce protease-dependent bistable dynamics, while the excitatory effect of ECM-neuronal interaction resulted in a richer repertoire of observable dynamical states. We found that for the excitatory ECM-neuron interactions, e.g. involving the inhibition of SK-channels or synaptic upscaling, the ECM concentration levels may exhibit different activity regimes, ranging from neural firing-induced protease-independent switching between stationary states of the ECM concentration to spontaneous ECM oscillations, which might coexist with a stationary concentration level. In terms of neuronal activity, this means that there are different dynamical modes of ultra-slow firing threshold modulation or modulation of the power of the synaptic scaling effect. Development of more detailed network-based models of neural activity subjected to these ultra-slow modulations might predict the functional effects by which changes in the ECM induced by a seizure or emotional stress might persistently alter the activity of neuronal circuits.

Obviously, the next step for the development of the model is to compare its predictions with experimentally observed dynamics of neuronal activity, activities of ECM proteases and expression of neural ECM components. Ca^2+^ imaging and multielectrode arrays can be used in vitro and in vivo to monitor neuronal activity. Live labeling of ECM of perineuronal nets with Vicia villosa agglutinin is possible in vitro to compare ECM expression at two time-points [18], but obviously this approach is far from ideal for quantitative analysis of ECM dynamics. Recently, we introduced adeno-associated viruses expressing fluorescently tagged mutated link protein Hapln1 for efficient neural ECM labeling in vitro and in vivo (not published). There are biosensors for MMP-9 proteolytic activity [19], as one of proteases involved in remodeling of neural ECM. However, MMP-9 seems to be not the major enzyme for remodeling of ECM and hence new biosensors for live imaging of aggrecanase activities against aggrecan and/or other members of the aggrecan family [20], should be first designed and validated before we can try to fit our models to experimental data. Also, more detailed description of neural networks with inhibitory and excitatory neurons, and more differentiated regulations of ECM associated with these cell types should be introduced in the model. It is surely worth to invest in these developments as the nature of ECM is such that rapid proteolytic degradation is followed by slower recovery of ECM, enabling dramatic long-term switches between ECM levels, as suggested by the present work. These changes in ECM may reopen the critical transition period for global readjustment of neural network. Analysis of adaptive and maladaptive values of these transitions may generate insights into pathogenesis of diverse brain diseases.
